# The TMPRSS2 Inhibitor Nafamostat Reduces SARS-CoV-2 Pulmonary Infection in Mouse Models of COVID-19

**DOI:** 10.1128/mBio.00970-21

**Published:** 2021-08-03

**Authors:** Kun Li, David K. Meyerholz, Jennifer A. Bartlett, Paul B. McCray

**Affiliations:** a Department of Pediatrics, University of Iowa, Iowa City, Iowa, USA; b Department of Pathology, University of Iowa, Iowa City, Iowa, USA; c Department of Microbiology and Immunology, University of Iowa, Iowa City, Iowa, USA; University of Maryland School of Medicine; Johns Hopkins Bloomberg School of Public Health

**Keywords:** COVID-19, SARS-CoV-2, MERS-CoV, nafamostat, camostat, TMPRSS2, serine protease inhibitors, airway epithelia, Ad5-*hACE2*, K18-*hACE2*, coronavirus, preclinical drug studies

## Abstract

The coronavirus disease 2019 (COVID-19) pandemic has caused significant morbidity and mortality on a global scale. The etiologic agent, severe acute respiratory syndrome coronavirus 2 (SARS-CoV-2), initiates host cell entry when its spike protein (S) binds to its receptor, angiotensin-converting enzyme 2 (ACE2). In airway epithelia, the spike protein is cleaved by the cell surface protease TMPRSS2, facilitating membrane fusion and entry at the cell surface. This dependence on TMPRSS2 and related proteases suggests that protease inhibitors might limit SARS-CoV-2 infection in the respiratory tract. Here, we tested two serine protease inhibitors, camostat mesylate and nafamostat mesylate, for their ability to inhibit entry of SARS-CoV-2 and that of a second pathogenic coronavirus, Middle East respiratory syndrome coronavirus (MERS-CoV). Both camostat and nafamostat reduced infection in primary human airway epithelia and in the Calu-3 2B4 cell line, with nafamostat exhibiting greater potency. We then assessed whether nafamostat was protective against SARS-CoV-2 *in vivo* using two mouse models. In mice sensitized to SARS-CoV-2 infection by transduction with human *ACE2*, intranasal nafamostat treatment prior to or shortly after SARS-CoV-2 infection significantly reduced weight loss and lung tissue titers. Similarly, prophylactic intranasal treatment with nafamostat reduced weight loss, viral burden, and mortality in K18-*hACE2* transgenic mice. These findings establish nafamostat as a candidate for the prevention or treatment of SARS-CoV-2 infection and disease pathogenesis.

## INTRODUCTION

Severe acute respiratory syndrome coronavirus 2 (SARS-CoV-2) is the causative agent of coronavirus disease 2019 (COVID-19) (1), a multiorgan syndrome characterized by severe pneumonia and additional gastrointestinal, cardiovascular, neurological, and systemic manifestations (2, 3). This novel coronavirus emerged in China in December 2019 and quickly reached pandemic status. As of 27 May 2021, the number of laboratory-confirmed infections has reached 168 million, resulting in nearly 3.5 million deaths worldwide (Johns Hopkins Coronavirus Resource Center; https://coronavirus.jhu.edu/). SARS-CoV-2 is evolutionarily related to SARS coronavirus (SARS-CoV), the etiologic agent of severe acute respiratory syndrome (SARS), which caused an epidemic of severe pneumonia in 2002 and 2003 (4). A second pathogenic member of the coronavirus family, Middle East respiratory syndrome coronavirus (MERS-CoV), emerged in 2012 (5, 6) and has caused over 850 deaths to date according to the World Health Organization.

Like SARS-CoV, SARS-CoV-2 utilizes the membrane ectopeptidase angiotensin-converting enzyme 2 (ACE2) as its receptor to initiate binding and entry (7). The virus engages ACE2 using the receptor binding domain of the spike glycoprotein (S). To facilitate fusion with host membranes, the S protein is “primed” by proteolytic cleavage events, first at the S1/S2 cleavage site and then at the S2′ site to liberate the N terminus of the fusion peptide on the S2 subunit. This entry process is cell type dependent. In tissue culture cell lines such as Vero E6, virions bind ACE2 at the cell surface, then enter an endosomal compartment where cathepsins mediate the S2′ cleavage and membrane fusion takes place (8). In contrast, in respiratory epithelia, the predominant mode of viral entry appears to be membrane fusion at the cell surface, where cell surface proteases such as TMPRSS2 execute the S2′ cleavage (7).

Studies in cultured human airway epithelia established that protease inhibitors targeting TMPRSS2 can block SARS-CoV-2 entry (as well as that of SARS-CoV and MERS-CoV), suggesting their therapeutic potential. One candidate is the serine protease inhibitor camostat mesylate, which was initially recognized for its ability to inhibit TMPRSS2-mediated membrane fusion and entry of influenza viruses in airway epithelia (9–11). Camostat reduced SARS-CoV-2 pseudovirion entry into the human lung cell line Calu-3 (7), and it has similar activity against SARS-CoV and MERS-CoV (12, 13). A related serine protease inhibitor, nafamostat mesylate, also potently inhibits SARS-CoV-2 and MERS-CoV *in vitro* (8, 14–17).

While these data are promising, experiments demonstrating protection against SARS-CoV-2 infection in animal models are lacking. Mice are not naturally permissive to SARS-CoV-2 infection, due to the low affinity of the S protein receptor binding domain for mouse Ace2. Previously, a transgenic mouse expressing the human *ACE2* gene under the control of the cytokeratin 18 (K18) promoter (the K18-*hACE2* mouse) was generated to study SARS-CoV pathogenesis (18). Recent studies have demonstrated that these mice also support SARS-CoV-2 infection and develop a lethal respiratory illness with weight loss, inflammation, and associated brain infection (19–22). Another model sensitizes mice to SARS-CoV-2 infection by adenoviral vector transduction to deliver a human *ACE2* transgene (Ad5-*hACE2*). Mice infected with SARS-CoV-2 under these conditions recapitulate features of COVID-19 lung disease, including pneumonia and severe lung pathology (23, 24).

Here, we tested the hypothesis that protease inhibitors can inhibit SARS-CoV-2 and MERS-CoV infection in primary human airway epithelial cells. Because nafamostat showed relatively greater potency in inhibiting coronavirus infection *in vitro*, we focused on nafamostat for *in vivo* efficacy studies. We found that nafamostat reduced lung tissue viral load and disease severity in two complementary mouse models of COVID-19. The inhibitory effects of nafamostat were dependent upon the time of administration and route of delivery, highlighting the importance of these features in clinical studies of COVID-19 prevention or treatment.

## RESULTS

### Camostat and nafamostat inhibit MERS-CoV and SARS-CoV-2 infection in well-differentiated human airway epithelial cells.

**FIG 1:**
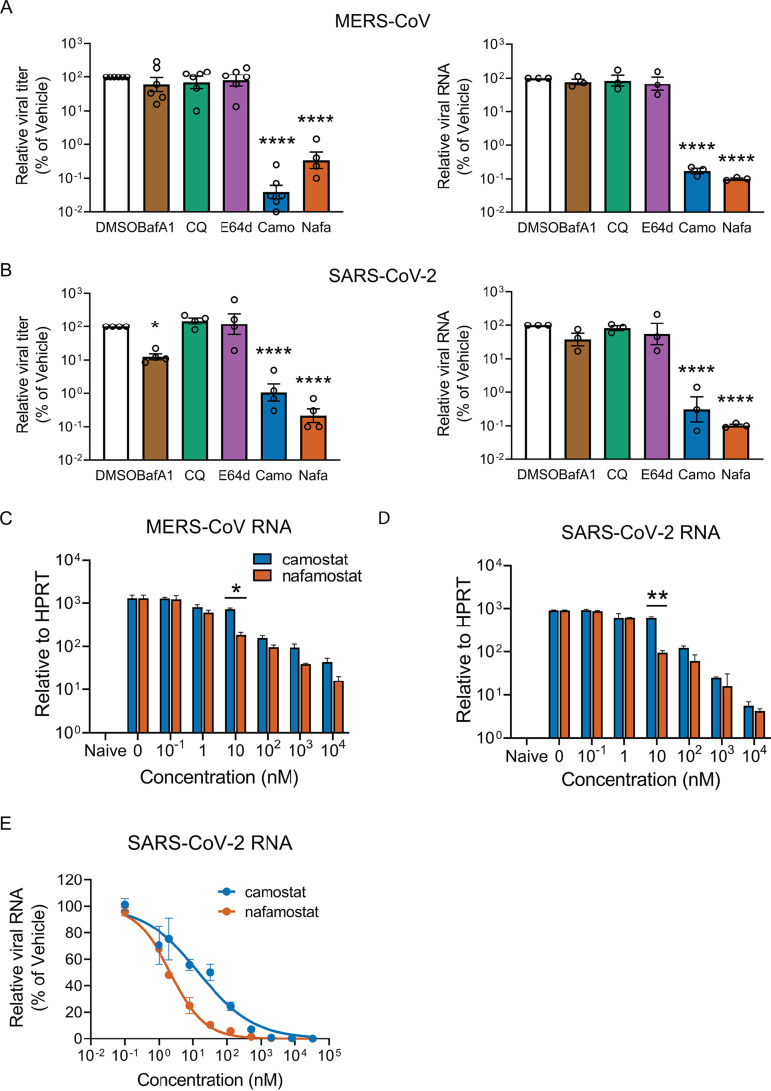
The serine protease inhibitors camostat and nafamostat potently inhibit MERS-CoV and SARS-CoV-2 replication in human airway epithelia. Primary human airway epithelia (HAE) were pretreated for 1 h with dimethyl sulfoxide (DMSO; vehicle), bafilomycin A1 (BafA1; 50 nM), chloroquine (CQ; 20 μM), E64d (25 μM), camostat (Camo; 25 μM), or nafamostat (Nafa; 25 μM), followed by infection with MERS-CoV (A) or SARS-CoV-2 (B) at a multiplicity of infection (MOI) of 0.1. At 20 h postinfection, apically released progeny virions were measured by plaque assay, and viral RNA levels were assessed by real-time quantitative PCR (qPCR), as described in Materials and Methods. Viral titers and RNA levels are expressed relative to those for infected cells with vehicle treatment, and data are presented as mean ± standard error (SE). Each data point represents an individual HAE donor. Log-transformed data were tested for significant differences from the vehicle control using one-way analysis of variance (ANOVA) followed by Dunnett’s multiple-comparison test. *, *P* < 0.05; ****, *P < *0.0001. (C, D) Calu-3 2B4 epithelial cells were preincubated in medium containing the indicated concentrations of camostat or nafamostat 1 h prior to infection. Cells were then infected with MERS-CoV or SARS-CoV-2 (MOI of 0.1) for 1 h and cultured overnight in medium containing the indicated inhibitor concentrations. At 20 h postinfection, viral RNA levels were quantified by real-time qPCR for MERS-CoV (C) or SARS-CoV-2 (D), as indicated. Data represent the mean 2^−Δ^*^CT^* ± SE (*C_T_*, threshold cycle). Log-transformed data were tested for statistically significant differences at each concentration using unpaired 2-tailed *t* tests, corrected for multiple comparisons by the Holm-Sidak method. *, adjusted (Adj.) *P < *0.05; **, Adj. *P < *0.01 (*n* = 3 replicate wells per condition). (E) Calu-3 2B4 cells were incubated with increasing concentrations of camostat or nafamostat 1 h prior to infection (MOI of 0.1), using the same procedure as shown in panels C and D. The reduction in SARS-CoV-2 RNA at 20 h postinfection was assessed by 2^−ΔΔ^*^CT^* method, using HPRT as a reference gene. Viral RNA levels are expressed relative to that for infected cells with vehicle treatment (*n* = 3 replicate wells per condition). Results represent two independent experiments.

We studied two coronaviruses that can cause serious disease in humans, SARS-CoV-2 and MERS-CoV. To better understand the protease requirements for entry of these two pathogenic coronaviruses in airway epithelia, we investigated their responses to several inhibitors during the early course of infection. We used primary cultures of well-differentiated human bronchial epithelia, which closely mimic the native surface airway epithelium *in vivo*. We tested camostat and nafamostat, bafilomycin A1 and chloroquine (both inhibitors of endosomal acidification), and E64d, an inhibitor of lysosomal cathepsins B and L. The inhibitors were added 1 h prior to infection with a multiplicity of infection (MOI) of 0.1 of each respective coronavirus, and 20 h later, we measured viral RNA abundance and the titer of virus released into airway surface liquid. In this setting, only camostat and nafamostat significantly inhibited infection of human airway epithelia (Fig. 1A and B). Thus, both MERS-CoV and SARS-CoV-2 depend on cell surface serine proteases such as TMPRSS2 for entry into primary airway epithelial cells. We found that the SARS-CoV-2 inhibitory activity of camostat and nafamostat were lost when infections were performed in TMPRSS2-negative Vero E6 cells, suggesting that these compounds likely work through inhibition of TMPRSS2 protease activity rather than through direct virucidal action (see Fig. S1 in the supplemental material).

10.1128/mBio.00970-21.1 FIG S1 Camostat and nafamostat do not inhibit SARS-CoV-2 infection in Vero E6 cells. Vero E6 cells were pretreated for 1 h with dimethyl sulfoxide (DMSO) (vehicle), E64d (25 μM), camostat (Camo; 25 μM), or nafamostat (Nafa; 25 μM), followed by infection with SARS-CoV-2 at a multiplicity of infection (MOI) of 0.1. At 24 h post infection, progeny virions in the medium were measured by plaque assay. Viral titer is expressed relative to that for infected cells with vehicle treatment, and data are presented as mean ± standard error (SE). Results represent 3 replicate experiments. Log-transformed data were tested for significant differences from the vehicle control using one-way ANOVA followed by Dunnett’s multiple-comparisons test. ****, *P* < 0.0001. Download FIG S1, TIF file, 0.1 MB. Copyright © 2021 Li et al. 2021 Li et al. https://creativecommons.org/licenses/by/4.0/ This content is distributed under the terms of the Creative Commons Attribution 4.0 International license.

### The camostat analogue nafamostat shows relatively greater potency against MERS-CoV and SARS-CoV-2.

Previous pseudovirus studies reported that nafamostat mesylate inhibits SARS-CoV-2 S protein mediated entry into Calu-3 cells with ∼15-fold higher efficiency than that of camostat mesylate, and that nafamostat blocks infection by authentic SARS-CoV-2 more effectively than camostat in these cells (14). We investigated camostat and nafamostat inhibition of authentic MERS-CoV or SARS-CoV-2 infection of Calu-3 2B4 cells (Fig. 1C to E). For both MERS-CoV (Fig. 1C) and SARS-CoV-2 (Fig. 1D), nafamostat pretreatment reduced viral RNA at 20 h postinfection more than camostat pretreatment. The 50% inhibitory concentration (IC_50_) for nafamostat was 2.2 nM (95% confidence interval [CI], 1.8 to 2.5 nM; *R*^2^ = 0.98) and for camostat was 14.8 nM (95% CI, 8.2 to 26.2 nM; *R*^2^ = 0.89) (Fig. 1E).

### Both intraperitoneal and intranasal nafamostat inhibit SARS-CoV-2 infection *in vivo*.

**FIG 2:**
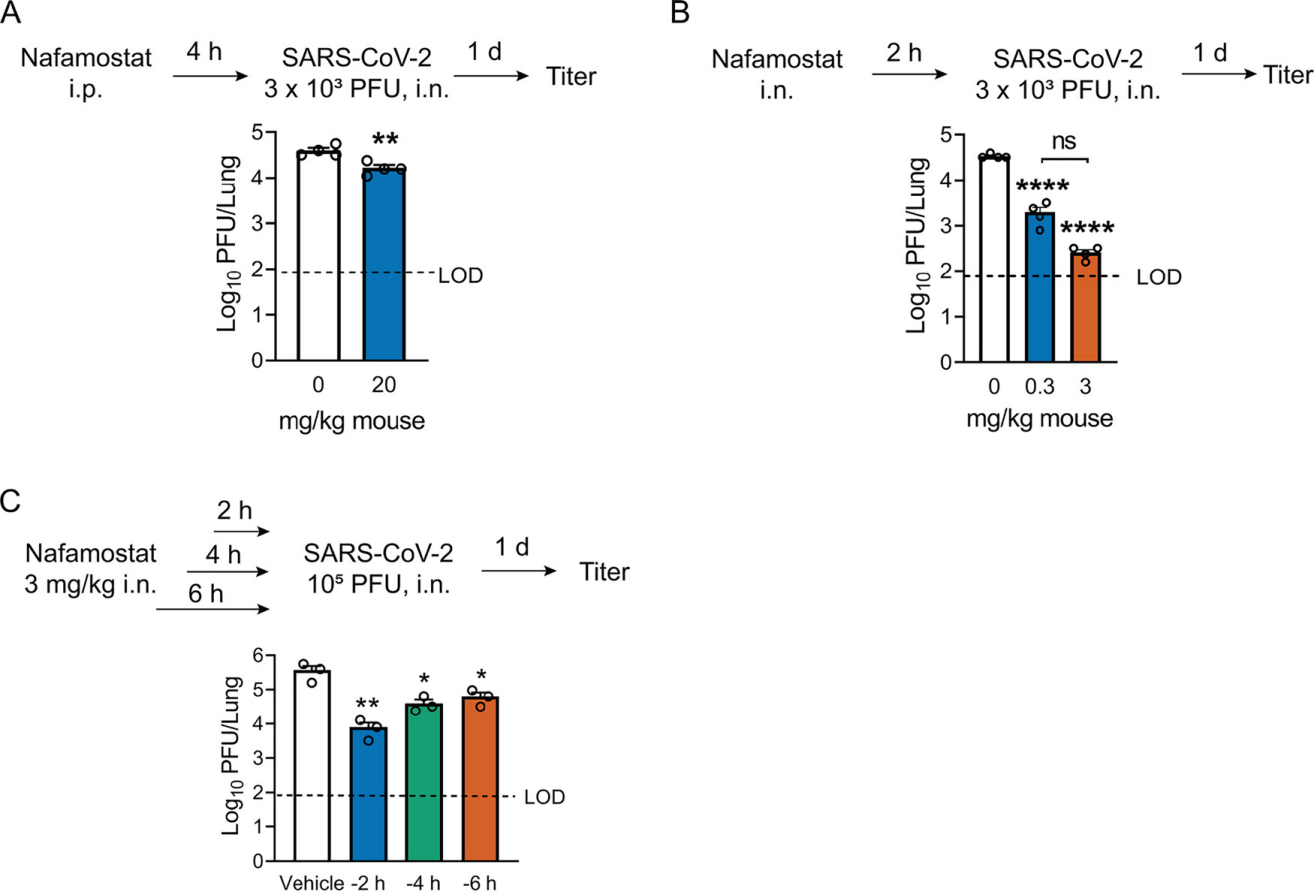
Efficacy of nafamostat in reducing lung tissue titers following SARS-CoV-2 infection. BALB/c mice were transduced intranasally (i.n.) with 2.5 × 10^8^ PFU of Ad5-*hACE2* as described in Materials and Methods. Five days later, (A) mice received 20 mg/kg nafamostat in 200 μl of PBS intraperitoneally (i.p.). Four hours later, mice were infected intranasally with 3 × 10^3^ PFU of SARS-CoV-2, and lung tissue virus titers were measured at 1 day postinfection. Data were tested for statistically significant differences by 2-tailed Student’s *t* test. (B) Ad5-*hACE2*-sensitized mice were treated intranasally with 0.3 mg/kg or 3 mg/kg nafamostat in 50 μl Dulbecco’s modified Eagle medium (DMEM) 2 h before intranasal infection with 3 × 10^3^ PFU of SARS-CoV-2. Lung tissue virus titers were measured at 1 day postinfection (*n* = 4 mice/group). (C) Ad5-*hACE2*-sensitized mice received 3 mg/kg nafamostat intranasally 2 h, 4 h, or 6 h prior to intranasal infection with SARS-CoV-2 (1 × 10^5^ PFU). Lung tissue virus titers were measured at 1 day postinfection (*n* = 3 mice/group). For results in panels B and C, data were tested for significant differences using one-way ANOVA followed by Tukey’s multiple-comparison test. In all panels, data are presented as mean ± SE. *, *P < *0.05; **, *P < *0.01; ****, *P < *0.0001. LOD, limit of detection. Each experiment was performed once.

Given its greater efficacy *in vitro*, we evaluated whether nafamostat could inhibit SARS-CoV-2 infection in a mouse model of COVID-19. Mice were sensitized to SARS-CoV-2 infection by intranasal transduction (Ad5-*hACE2*) (23, 24). Nafamostat was delivered via intraperitoneal (i.p.) or intranasal (i.n.) routes, and mice were inoculated intranasally with SARS-CoV-2. Nafamostat pretreatment inhibited SARS-CoV-2 infection more potently when delivered via the i.n. route. Intranasal nafamostat administration resulted in a nearly 2-log reduction in lung tissue viral titers at the highest dose tested (3 mg/kg) compared to a less than 5-fold reduction following i.p. (20 mg/kg) delivery (Fig. 2A and B). Nafamostat reduced titers when administered 2, 4, or 6 h prior to viral inoculation, with moderately better effectiveness if delivered nearer the time of SARS-CoV-2 infection (Fig. 2C).

### Nafamostat inhibits weight loss and virus burden in SARS-CoV-2-challenged mice.

**FIG 3:**
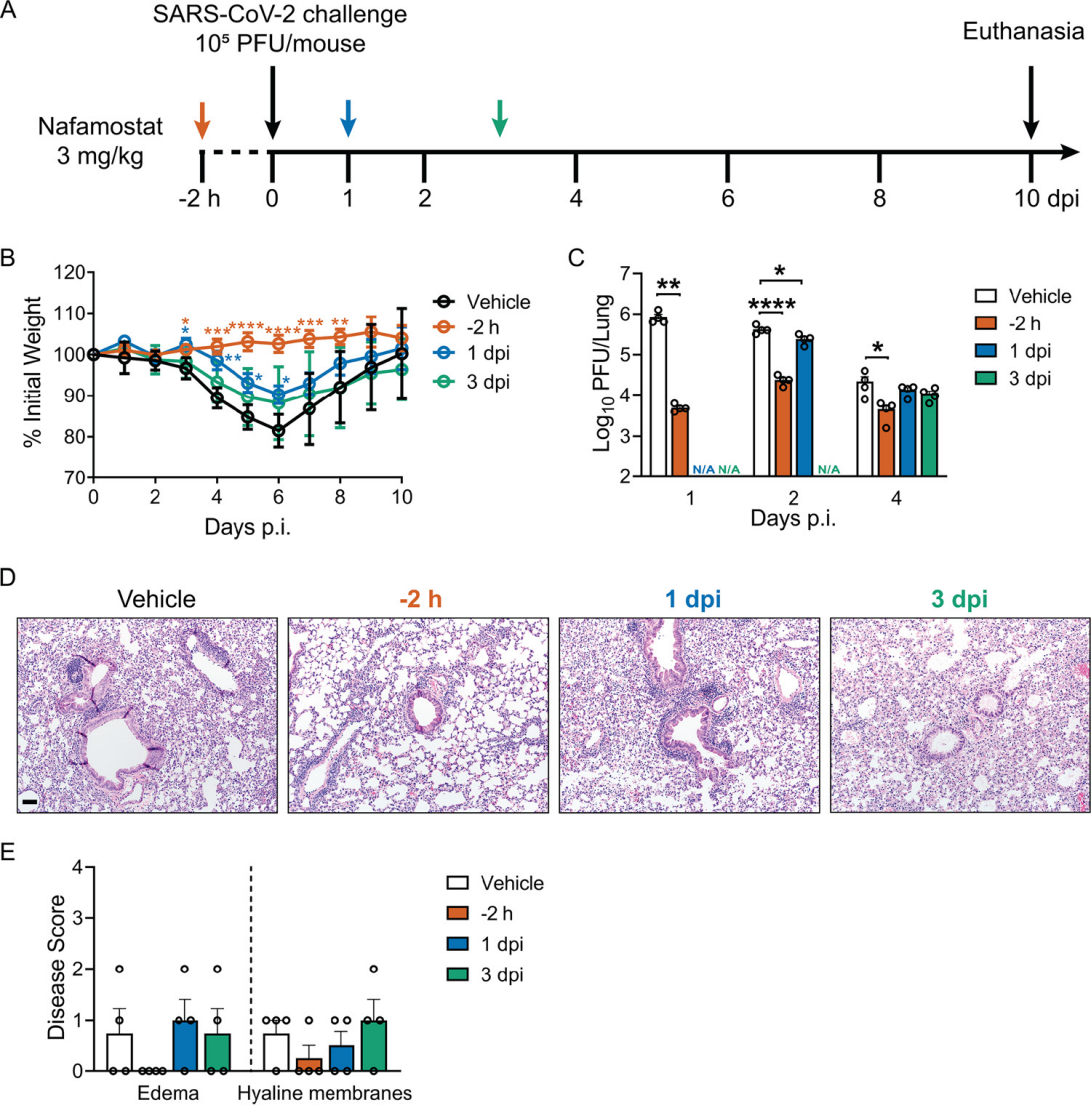
Single-dose intranasal treatment with nafamostat protects mice from SARS-CoV-2 infection. (A) Experimental protocol. Ad5-*hACE2*-transduced BALB/c mice were infected intranasally with 10^5^ PFU SARS-CoV-2 and treated via the i.n. route with vehicle or 3 mg/kg nafamostat at −2 h, 1 day, or 3 days postinfection. (B) Weight loss was monitored daily (*n* = 8 or 9 mice per group), and data were tested for significant differences using 2-way ANOVA followed by Dunnett’s posttest. Weight loss data represent results from two independent experiments. (C) Lung tissue titers quantified at 1, 2, and 4 days postinfection (N/A, not analyzed). Data were tested for significant differences using one-way ANOVA followed by Dunnett’s multiple-comparison test. *, *P < *0.05; **, *P < *0.01; ***, *P < *0.001; ****, *P < *0.0001. (D) Representative images of hematoxylin- and eosin-stained lung sections and (E) histopathologic scores at 5 days postinfection. Bar, 60 μm. Results represent 4 mice per group. In all panels, data are presented as mean ± SE. Data presented in panels C, D, and E represent one experiment.

To assess whether i.n. nafamostat administration altered the course of SARS-CoV-2 infection, mice were transduced with Ad5-*hACE2*, followed by i.n. infection with SARS-CoV-2 (10^5^ PFU/mouse). Animals received nafamostat (3 mg/kg, i.n.) at 2 h prior to infection, 1 day postinfection, or 3 days postinfection, and were monitored daily for weight loss (Fig. 3A). Nafamostat pretreatment abrogated SARS-CoV-2-induced weight loss (Fig. 3B). Weight loss was also significantly reduced in animals receiving nafamostat at 1 day postinfection (Fig. 3B). Consistent with these findings, lung viral loads were reduced at 1, 2, and 4 days postinfection in mice pretreated with nafamostat, whereas the reductions in lung viral titers were more modest in mice receiving nafamostat after SARS-CoV-2 challenge (Fig. 3C). Histopathological analysis of lung tissue from infected animals at 5 days postinfection suggests that nafamostat treatment reduced lung pathology in the infected mice, primarily in the animals receiving the pretreatment protocol (Fig. 3D and E). These results indicate that in mice expressing hACE2 via Ad5-*hACE2* transduction, nafamostat reduces SARS-CoV-2 infection severity, particularly when administered prior to or early in infection.

### Nafamostat protects against SARS-CoV-2 infection in K18-*hACE2* mice.

**FIG 4:**
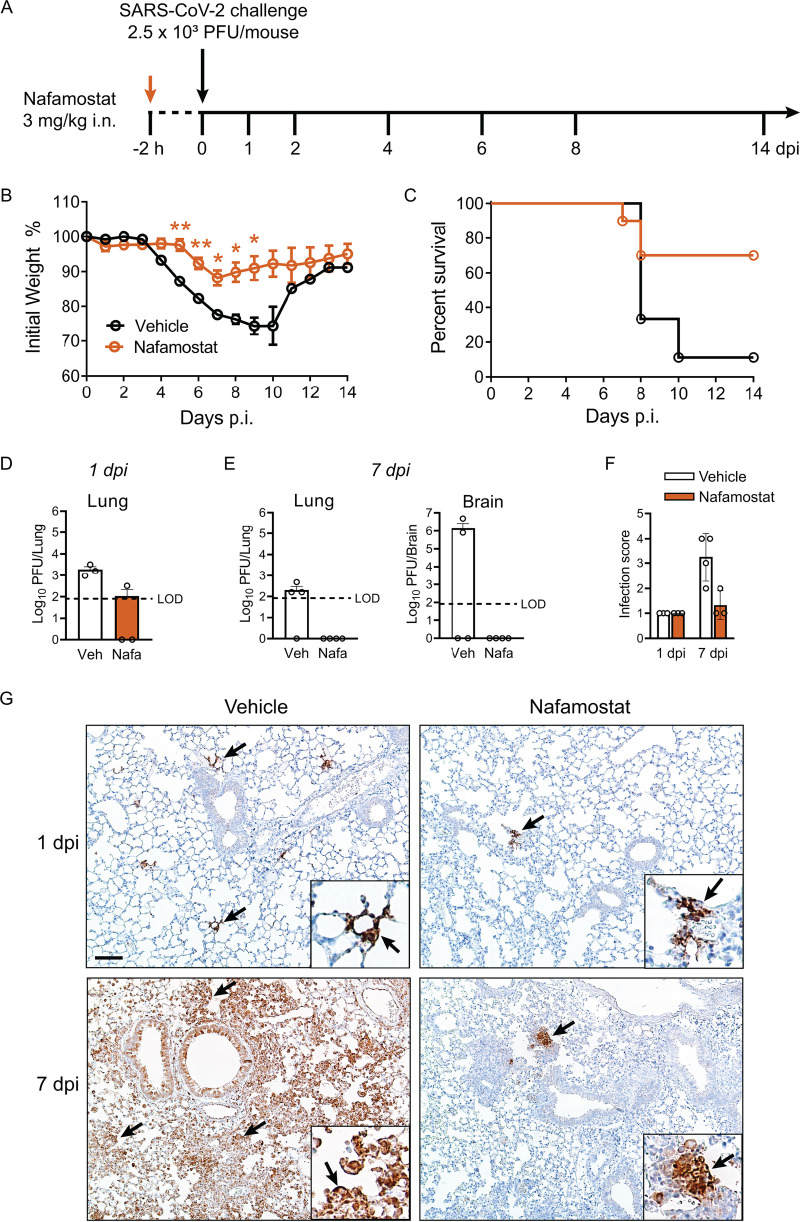
Nafamostat protects K18-*hACE2* mice from infection with SARS-CoV-2. (A) Schematic of experimental protocol. K18-*hACE2* mice were treated with i.n. nafamostat (3 mg/kg), and 2 h later they were infected i.n. with SARS-CoV-2 (2.5 × 10^3^ PFU/mouse). (B) Weight loss was monitored daily in nafamostat- and vehicle-treated mice (*n* = 9 mice/group), and data were tested for significant differences at each day postinfection using unpaired 2-tailed *t* tests, corrected for multiple comparisons by the Holm-Sidak method. *, Adj. *P < *0.05; **, Adj. *P < *0.01 (C) Survival curves for nafamostat- and vehicle-treated mice. Weight loss and survival curve data represent results from two independent experiments. (D) Lung tissue virus titers measured at 1 day postinfection. No virus was detected in the brain for either treatment group at 1 day postinfection (*n* = 3 mice/group). LOD, limit of detection. (E) Virus titers in the lungs and brain 7 days postinfection (*n* = 4 mice/group). (F) Immunohistochemistry identified SARS-CoV-2-infected cells in lung tissue sections from vehicle-and nafamostat-treated mice at 1 and 7 days postinfection. Tissues were stained for the SARS-CoV-2 N protein (brown) and scored as described in Materials and Methods. Significant differences between vehicle- and nafamostat-treated mice at each time point were assessed by the Mann-Whitney test. In all panels, data are presented as mean ± SE. (G) Representative images of lung tissue from vehicle- and nafamostat-treated K18-*hACE2* mice at 1 and 7 days postinfection, immunostained for SARS-CoV-2 N protein (black arrows). Bar, 92 μm. Data presented in panels D to G represent one experiment.

We next tested nafamostat in K18-*hACE2* mice (18). K18-*hACE2* mice were pretreated with i.n. nafamostat (3 mg/kg) for 2 h, followed by SARS-CoV-2 challenge (2.5 × 10^3^ PFU/mouse) (Fig. 4A). Over a 14-day time course, nafamostat-treated mice lost less weight and exhibited significantly less mortality than vehicle-treated controls (Fig. 4B and C). At 1 day postinfection, virus was detected in the lungs of vehicle-treated mice but was largely undetectable in tissue from nafamostat-treated mice (Fig. 4D). By 7 days postinfection, virus titers could be measured in both lung and brain in 50% of vehicle-treated mice, whereas no virus was detected in lung or brain tissue from mice receiving nafamostat (Fig. 4E).

These results suggest that nafamostat pretreatment significantly reduced viral loads over the course of SARS-CoV-2 infection. We examined the distribution of SARS-CoV-2-positive cells in tissues from K18-*hACE2* mice by immunostaining for viral antigen. In the lungs of vehicle-treated animals, infection was widespread throughout the cells of the small airways and alveoli by 7 days postinfection. In contrast, SARS-CoV-2-positive cells were far less abundant (though not entirely absent) in tissue from nafamostat-treated mice (Fig. 4F and G). Viral infection in the brain was more variable. While there were no virus-positive cells in either treatment group at 1 day postinfection, by 7 days postinfection, at least half of the vehicle-treated animals exhibited profound brain infection (see Fig. S2 in the supplemental material), generally mirroring the virus tissue titers. This finding of later onset of brain infection was previously reported (19, 21, 22). Infected cells were also found in the sinonasal cavity, with nafamostat-treated mice showing a trend toward fewer SARS-CoV-2-positive cells in the maxillary sinus and olfactory epithelium (Fig. S2). Very few lung lesions were observed in either treatment group (see Fig. S3 in the supplemental material), making it difficult to assess whether nafamostat treatment reduced lung disease severity in K18-*hACE2* mice.

10.1128/mBio.00970-21.2 FIG S2 SARS-CoV-2 infection in brain and sinonasal tissues in vehicle- and nafamostat-treated K18-*hACE2* mice. Vehicle- and nafamostat-treated K18-*hACE2* mice were infected with SARS-CoV-2, and tissues were harvested at 1 and 7 days postinfection. Infection was assessed in the brain (A to D), maxillary sinus (E to H), and olfactory epithelium (I to L), by immunohistochemical staining (brown) for the SARS-CoV-2 N protein (black arrows). Viral infection was compared for vehicle-treated (A, C, E, G, I, K) versus nafamostat-treated (B, D, F, H, J, L) mice, at 1 (A, B, E, F, I, J) and 7 days (C, D, G, H, K, L) postinfection. For brain images (A to D), bar = 460 μm. For all other panels, bar = 92 μm. Download FIG S2, TIF file, 2.5 MB. Copyright © 2021 Li et al. 2021 Li et al. https://creativecommons.org/licenses/by/4.0/ This content is distributed under the terms of the Creative Commons Attribution 4.0 International license.

10.1128/mBio.00970-21.3 FIG S3 Histopathology in lungs from vehicle- and nafamostat-treated K18-*hACE2* mice after SARS-CoV-2 infection. Shown are representative images of hematoxylin- and eosin-stained lung tissue sections from vehicle- and nafamostat-treated K18-*hACE2* mice at 1 and 7 days post SARS-CoV-2 infection. Bar, 230 μm. Download FIG S3, TIF file, 2.4 MB. Copyright © 2021 Li et al. 2021 Li et al. https://creativecommons.org/licenses/by/4.0/ This content is distributed under the terms of the Creative Commons Attribution 4.0 International license.

## DISCUSSION

Here, we show that the serine protease inhibitors camostat and nafamostat potently reduce SARS-CoV-2 and MERS-CoV infection in well-differentiated primary cultures of airway epithelia, presumably by inhibiting the activity of cell surface serine proteases (such as TMPRSS2). Both camostat and nafamostat were previously shown to directly inhibit the enzymatic activity of TMPRSS2 and related serine proteases in biochemical assays (25), strongly suggesting that inhibition of TMPRSS2 catalytic activity is the primary mechanism for this effect. In contrast, we saw little or no effect from bafilomycin A1, chloroquine, or E64d, agents that alter pH and/or protease function in intracellular compartments, including endosomes and lysosomes. Our results agree with those of earlier studies (7, 8, 13–17, 26, 27) and contribute to the growing consensus that fusion and entry at the plasma membrane is the preferred route of entry into cells of the respiratory tract. Our experiments in Calu-3 2B4 cells indicate that while both camostat and nafamostat are active against SARS-CoV-2 and MERS-CoV, nafamostat is more potent, a trend also observed in other *in vitro* studies (14, 15).

Importantly, we demonstrate the *in vivo* efficacy of nafamostat in reducing SARS-CoV-2 infection and pathogenesis. The protective effect of nafamostat was greatest when the drug was administered prior to viral infection. Pretreatment with nafamostat via the i.n. route either completely prevented or significantly reduced infection-induced weight loss, and substantially reduced viral loads throughout the ensuing course of illness, in Ad5-*hACE2* transduced mice and K18-*hACE2* mice. In Ad5-*hACE2* transduced mice, although nafamostat treatment beginning at 1 day postinfection provided some protection, the results were less dramatic. These findings suggest that protease inhibitor treatment may provide its greatest clinical benefit when delivered prophylactically or in the early stages of infection, and that there may be a “treatment window” after which treatment no longer improves outcomes. We note that the course of SARS-CoV-2 infection in hACE2-expressing mice proceeds more rapidly and on a shorter time course than in humans.

Further highlighting the importance of timing for the *in vivo* efficacy of nafamostat, we observed that the effectiveness of intranasal nafamostat increased as the time interval between nafamostat delivery and viral inoculation decreased (Fig. 2C). This likely reflects the relatively short half-life of nafamostat; early studies with nafamostat reported a plasma half-life of 8 min in rabbits and 1 min in dogs (28). Currently, there are no data regarding nafamostat stability in respiratory secretions following i.n. administration. It is possible that the fate of nafamostat is different in airway secretions than that in plasma, potentially contributing to the different outcomes observed via i.n. or i.p. routes in our study. It is also unknown how efficiently nafamostat is transported into airway secretions when delivered systemically, which may influence outcomes following i.n. versus i.p. administration. Pharmacokinetic studies are needed to better understand these aspects of nafamostat activity.

Based on their encouraging *in vitro* activity against SARS-CoV-2, both camostat and nafamostat are currently under evaluation as potential therapies for COVID-19 (https://clinicaltrials.gov; NCT04652765, NCT04455815, NCT04353284, NCT04583592, NCT04470544, NCT04435015, NCT04608266, NCT04524663, NCT04750759, NCT04625114, NCT04730206, NCT04321096, NCT04355052, NCT04662073, NCT04681430, NCT04644705, NCT04657497, NCT04374019, NCT04418128, NCT04352400, NCT04390594, NCT04628143, NCT04623021, NCT04473053, NCT04483960). Both compounds are approved treatments for other medical conditions and are therefore attractive candidates for rapid drug repurposing. In Japan, camostat is approved for use in treatment of acute pancreatitis and postoperative reflux esophagitis (29), and it has a well-characterized safety profile. Camostat treatment was shown to improve survival in a mouse model of SARS-CoV infection (30). Nafamostat is marketed in Japan and South Korea for treatment of acute pancreatitis and disseminated intravascular coagulation (DIC). *In vitro* studies indicate that nafamostat does not cause cytotoxicity in cultured human endothelial or airway epithelial cells (14, 31–33), and a recent case report describing nafamostat administration in three elderly COVID-19 patients reported no adverse events (34). The proposed clinical trials generally involve systemic administration of nafamostat. In our studies with the Ad5-*hACE2* mice, we observed that nafamostat reduced infection more effectively when delivered via the i.n. route, suggesting that it may be important to consider the route of administration when designing treatment regimens with protease inhibitors in human patients. It is possible that nafamostat’s therapeutic efficacy might be boosted by direct delivery to the airways as a nasal spray or inhaled aerosol. It is of note that nafamostat is a broad-spectrum protease inhibitor with effects on multiple biological processes, which has led to speculation that it may confer benefits beyond blocking SARS-CoV-2 entry. In particular, its anticoagulant properties may reduce or prevent COVID-19-related thrombotic complications. Nafamostat also inhibits proteases involved in inflammatory cascades and the complement system, which may dampen inflammation in severe COVID-19 cases.

In conclusion, we provide evidence that camostat and nafamostat potently inhibit SARS-CoV-2 and MERS-CoV infection in cultured human airway epithelia; nafamostat exhibited greater potency than camostat in reducing SARS-CoV-2 infection, suggesting that it may be a more attractive candidate for COVID-19 lung disease prevention or treatment. Nafamostat inhibited SARS-CoV-2 infection and improved disease outcomes in two COVID-19 mouse models. Our experiments in these animal models highlight the importance of route and timing of administration in the design of effective treatment regimens. These preclinical data support further investigation of protease inhibitors as antiviral prophylactic or therapeutic strategies for COVID-19.

## MATERIALS AND METHODS

### Inhibitors, chemicals, and viruses.

Camostat mesylate, nafamostat mesylate, bafilomycin A, chloroquine, and E64d were purchased from the Sigma-Aldrich Corporation (St. Louis, MO). The EMC/2012 strain of MERS-CoV was provided by Bart Haagmans and Ron Founchier (Erasmus Medical Centre, Rotterdam Netherlands). The USA-WA1/2020 strain of SARS-CoV-2 was obtained from the BEI Resources Repository (https://www.niaid.nih.gov/research/bei-resources-repository; catalog no. NR-52281).

### Cell culture.

Primary human airway epithelia were prepared from bronchi as previously described (35). Briefly, epithelial cells were dissociated and seeded onto collagen-coated, semipermeable membranes with a 0.4-μm pore size (Costar Transwell, surface area, 0.33 cm^2^; Corning) in 24-well plates maintained in Ultroser G (USG) medium at 37°C and 5% CO_2_. At 24 hours after seeding, the mucosal medium was removed, and cells were grown at the air-liquid interface. Only well-differentiated cultures (>3 weeks old; resistance, >1,000 Ω · cm^2^) were used in this study. Calu-3 2B4 cells were maintained in minimal essential medium (MEM) supplemented with 20% fetal bovine serum (FBS), 0.1 mM nonessential amino acids (NEAA), 1 mM sodium pyruvate, 2 mM l-glutamine, 1% penicillin and streptomycin, and 0.15% NaHCO_3_ at 37°C with 5% CO_2_. Vero E6 cells were grown in Dulbecco’s modified Eagle medium (DMEM) supplemented with 10% FBS, 0.1 mM NEAA, and 1% penicillin and streptomycin at 37°C in 5% CO_2_.

### Infections in airway epithelia.

To investigate proteases required for MERS-CoV or SARS-CoV-2 infection in primary human airway epithelia, cells were incubated in medium (50 μl in the apical compartment and 500 μl basolaterally) containing bafilomycin A1 (50 nM), chloroquine (20 μM), E64d (25 μM), camostat (25 μM), or nafamostat (25 μM) at 37°C for 1 h. After 1 h of pretreatment, the apical medium was removed and replaced by medium containing MERS-CoV or SARS-CoV-2 (MOI of 0.1) in the presence of the indicated inhibitor/chemical for another 1 h incubation. The apical medium (containing unbound virus) was then removed, and cells were washed 2 times with phosphate-buffered saline (PBS) at the apical surface. At 20 h postinfection, the apical surface of infected cultures was rinsed with PBS to collect airway surface liquid (ASL), and titer was determined to verify the release of progeny virions into the ASL. Total cellular RNA was harvested in TRIzol reagent (Invitrogen, Waltham, MA).

To compare the efficacy of camostat and nafamostat against MERS-CoV or SARS-CoV-2 infection, Calu-3 2B4 cells were cultured in 96-well plates and pretreated with the indicated concentrations of inhibitors for 1 h. Cells were then infected with MERS-CoV or SARS-CoV-2 (MOI = 0.1) in the presence of the inhibitors for 1 h, followed by overnight incubation with the inhibitor. Total cellular RNA was harvested in TRIzol reagent at 20 h postinfection.

### Transduction and infection of Ad5-*hACE2* mice.

Ad5-*hACE2* was generated by the University of Iowa Viral Vector Core Facility. Six- to eight-week-old BALB/c mice were lightly anesthetized with ketamine-xylazine and transduced via the i.n. route with 2.5 × 10^8^ PFU of Ad5-*hACE2* in 75 μl DMEM. At 5 days postransduction, mice were infected i.n. with SARS-CoV-2 (3 × 10^3^ or 1 × 10^5^ PFU, as indicated). To make a stock of nafamostat for *in vivo* studies, the compound was dissolved in H_2_O at a concentration of 10 mg/ml. This nafamostat stock (or H_2_O for vehicle control animals) was diluted in PBS prior to i.p. injection; for i.n. delivery, the nafamostat stock was diluted in DMEM and delivered as a liquid bolus in a total volume of 50 μl. After SARS-CoV-2 infection, mice were monitored and weighed daily. All work with SARS-CoV-2 was conducted in the biosafety level 3 (BSL3) Laboratory of the University of Iowa. All protocols were approved by the Institutional Animal Care and Use Committees of the University of Iowa.

### Experiments with K18-*hACE2* mice.

Transgenic mice expressing human ACE2 under the control of the cytokeratin 18 promoter were previously reported (18). The 6- to 8-week-old mice used in these studies were obtained from the Jackson Laboratory [034860-B6.Cg-Tg(K18-ACE2)2Prlman/J] and are congenic on the C57BL/6 background.

### SARS-CoV-2 plaque assay.

Viral preps and lung or brain homogenate supernatants were serially diluted in DMEM. Vero E6 cells in 12-well plates were inoculated at 37°C in 5% CO_2_ for 1 h with gentle rocking every 15 min. After removing the inoculum, wells were overlaid with 1.2% agarose containing 4% FBS. After further incubation for 3 days, overlays were removed, and plaques were visualized using 0.1% crystal violet stain. Viral titers were calculated as PFU per lung or brain, as indicated.

### Quantitative real-time PCR analysis of viral RNA.

Total cellular RNA was isolated using the Direct-zol RNA miniprep kit (Zymo Research, Irvine, CA) following the manufacturer’s protocol and including a DNase treatment step. Total RNA (200 ng) was used as the template for first-strand cDNA synthesis. The resulting cDNA was used to quantify the MERS-CoV or SARS-CoV-2 RNA levels by real-time quantitative PCR using Power SYBR green PCR master mix (Applied Biosystems, Waltham, MA). Average values from duplicates of each sample were used to calculate the viral RNA level relative to the HPRT gene and presented as 2^−Δ^*^CT^* or 2^−ΔΔ^*^CT^*, as indicated (where *C_T_* is the threshold cycle). The primers used were as follows: MERS-CoV-F, 5′-CCACTACTCCCATTTCGTCAG-3′, and MERS-CoV-R, 5′-CAGTATGTGTAGTGCGCATATAAGCA-3′; 2019-nCoV-F, 5′-GACCCCAAAATCAGCGAAAT-3′, and 2019-nCoV-R, 5′-TCTGGTTACTGCCAGTTGAATCTG-3′; and hHPRT-F, 5′-AGGATTTGGAAAGGGTGTTTATTC-3′, and hHPRT-R, 5′-CAGAGGGCTACAATGTGATGG-3′.

### Histology and immunohistochemistry.

Mice were anesthetized and perfused transcardially with PBS. Tissues (lungs, brain, and nasal cavity) were harvested and fixed in 10% neutral buffered formalin (for 7 days), nasal cavities were decalcified in EDTA, and then all tissues were dehydrated through a series of alcohol and xylene baths, paraffin embedded, sectioned at ∼4 μm, and stained with hematoxylin and eosin (HE) stain. Serial sections were immunostained using a rabbit monoclonal antibody (catalog no. 40143-R019; Sino Biological, Beijing, China) against SARS-CoV-2 N protein, as previously described (22). Tissues were examined by a board-certified veterinary pathologist using a postexamination method of masking and following principles for reproducible tissue scores (36). Immunostaining of SARS-CoV-2 infection in the lung was scored using distribution-based ordinal scores: 0, absent; 1, <25%; 2, 26 to 50%; 3, 51 to 75%; and 4, >75% of lung fields.

### Statistical analysis.

Results are reported as mean ± standard error (SE). Data were tested for significant differences using Student’s *t* test, the Mann-Whitney test, and one-way analysis of variance (ANOVA) followed by Tukey’s or Dunnett’s tests of multiple comparisons, or by 2-way ANOVA followed by Dunnett’s or Sidak’s posttests, as indicated. All statistical tests were performed using GraphPad Prism 7. *P* values of <0.05 were considered statistically significant (*, *P < *0.05; **, *P* < 0.01; ***, *P* < 0.001; ****, *P* < 0.0001).
