# Supplementary figures and images for: The TMPRSS2 Inhibitor Nafamostat Reduces SARS-CoV-2 Pulmonary Infection in Mouse Models of COVID-19
Source: mBio. 2021 Aug 3;12(4):e00970-21. doi: 10.1128/mBio.00970-21 (PMC8406266; doi:10.1128/mBio.00970-21)

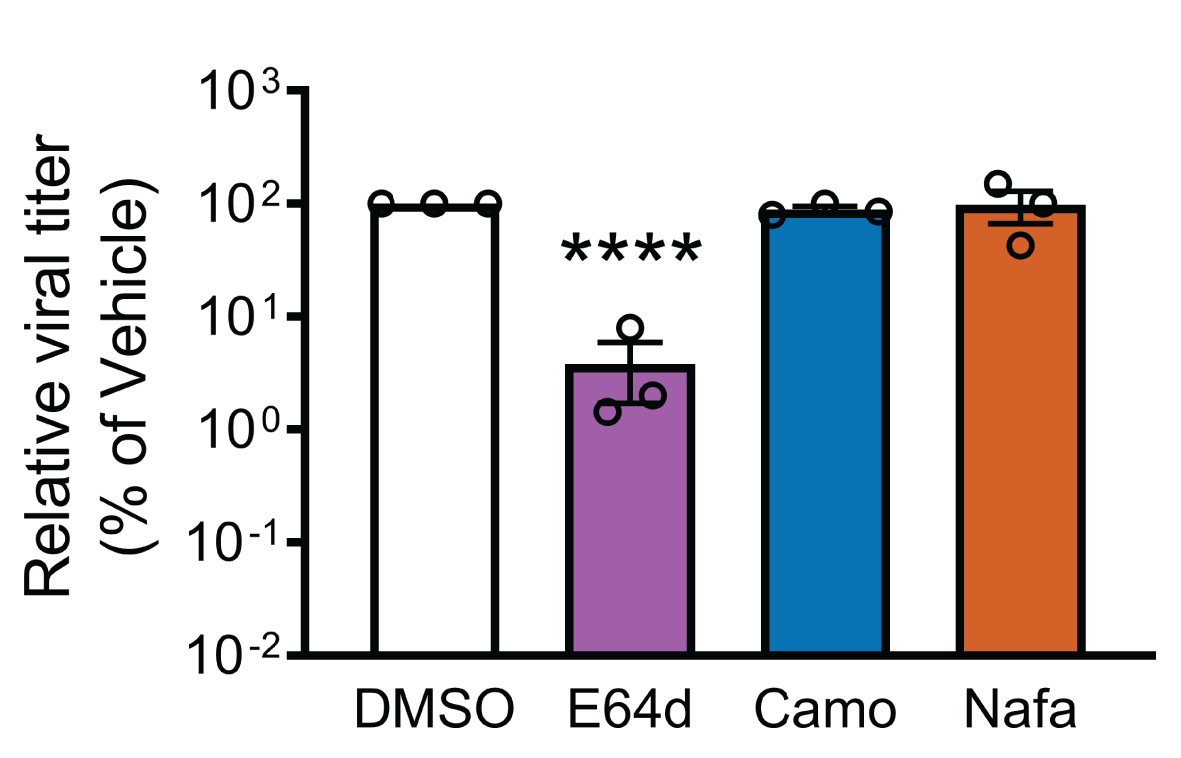

Supplement: FIG S1 [file mbio.00970-21-sf001.tif]

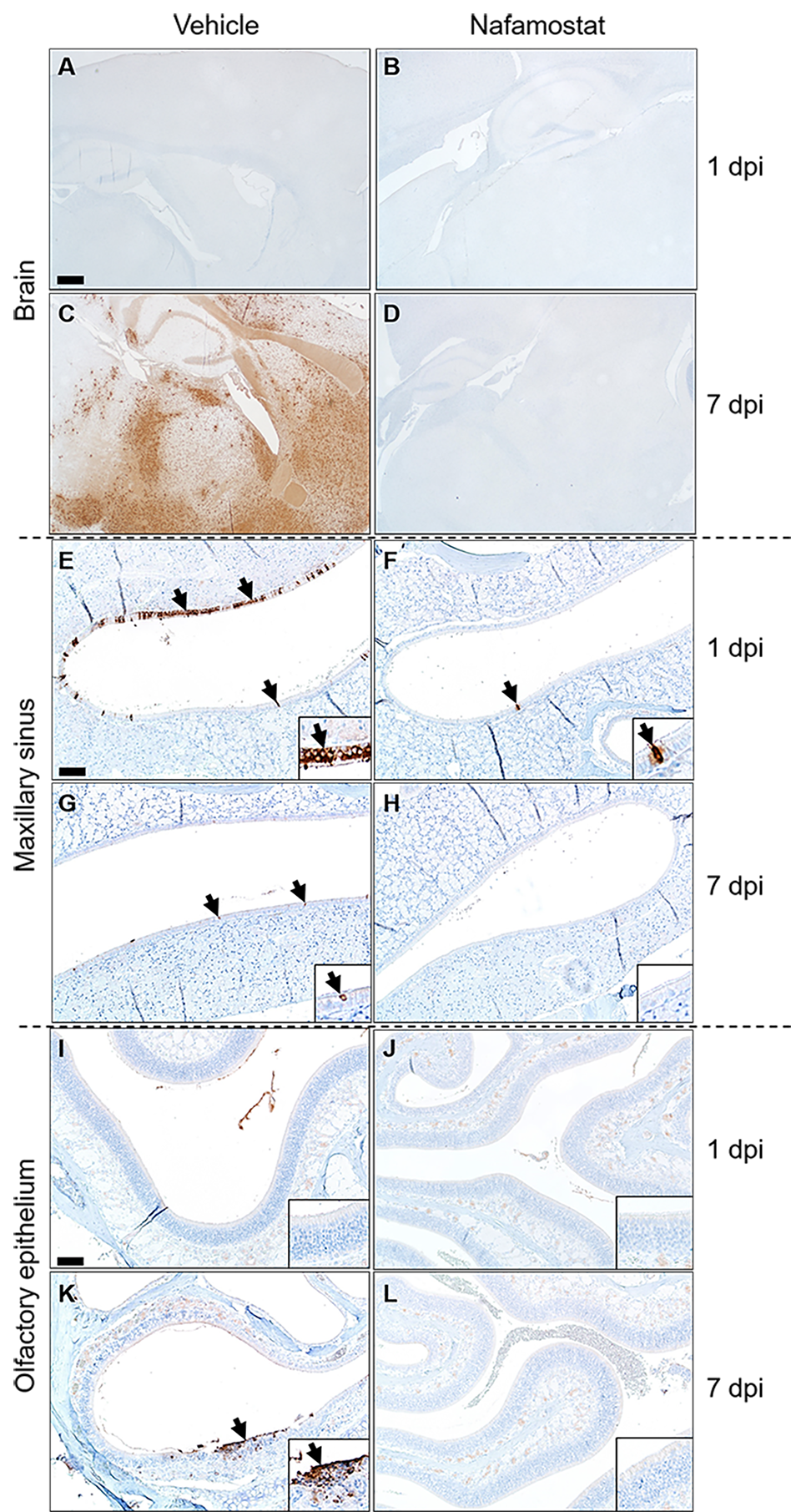

Supplement: FIG S2 [file mbio.00970-21-sf002.tif]

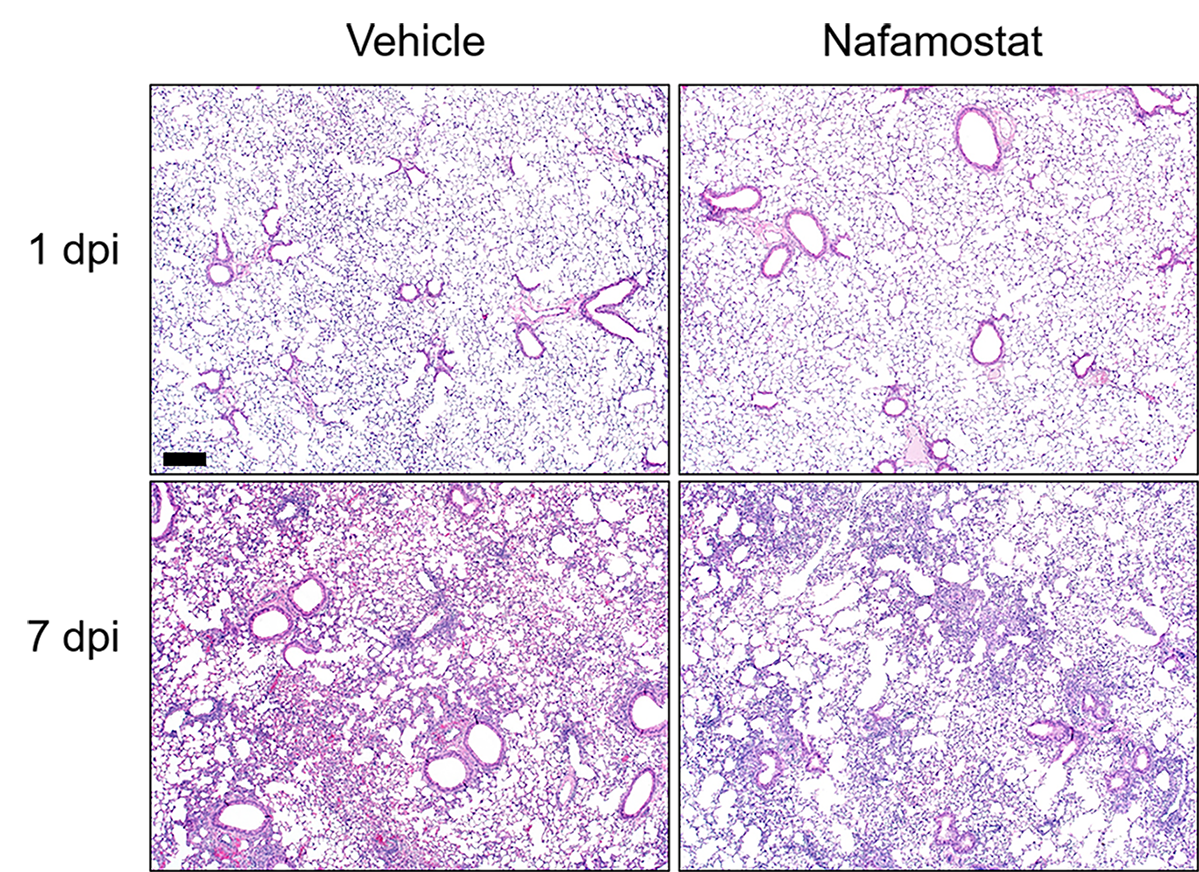

Supplement: FIG S3 [file mbio.00970-21-sf003.tif]
